# The Involvement of Melatonin in the Dimorphism of Glucose and Lipid Metabolism of Tilapia

**DOI:** 10.3390/biom16010015

**Published:** 2025-12-21

**Authors:** Jingkai Qin, Chenguang Liu, Zongzhen Liao, Yang Yu, Caiyun Sun, Wensheng Li

**Affiliations:** State Key Laboratory of Biocontrol, Guangdong Province Key Laboratory for Aquatic Economic Animals, Guangdong Provincial Engineering Technology Research Center for Healthy Breeding of Important Economic Fish, School of Life Sciences, Sun Yat-Sen University, Guangzhou 510275, China; qinjk@alumni.sysu.edu.cn (J.Q.); scoge84092@smc.edu.kg (C.L.); liaozzh@mail2.sysu.edu.cn (Z.L.); yuyang65@mail2.sysu.edu.cn (Y.Y.); suncaiy@mail.sysu.edu.cn (C.S.)

**Keywords:** aquaculture, gene expression, growth axis, metabolomics, *Oreochromis niloticus*, pathway analysis, sexual dimorphism

## Abstract

In tilapia, males grow faster than females, but the metabolites/pathways behind this sexual dimorphism remain unclear. In this study, we first examined growth, feeding, serum biochemical parameters, and mRNA expression in tilapia under mono-sex and mixed-sex cultures. Tilapia raised mono-sexually grow faster than those raised in mixed-sex environments. We conducted a combined analysis of the gas chromatography–mass spectrometry (GC-MS)-based metabolome and digital gene expression (DGE) profile in the livers of immature and mature tilapia, with special reference to the sexual differences. The glucose and lipid metabolism pathways exhibited significant sexual dimorphism. The concentrations of melatonin, oxoadipate, and glucuronic acid in the tryptophan metabolism pathway showed sexual differences. Melatonin implantation inhibited food intake and growth in tilapia and impacted their glucose and lipid metabolism. After melatonin implantation, the glucose tolerance of tilapia improved, especially in females. This study generates comprehensive data elucidating the mechanisms underlying sexual dimorphism in glucose and lipid metabolism and establishes a reliable scientific basis for investigating the role of melatonin in the sexual dimorphism of tilapia.

## 1. Introduction

Sexual growth dimorphism (SGD) is a phenomenon in which individuals of one sex in the same species grow faster than the other, which is common in animals. SGD is not only reflected in phenotypic differences, such as body mass and body composition, but also in physiological regulation.

The growth of animals is the process of converting ingested substances and energy into body components, which is closely related to the regulation of the growth axis, food intake, and metabolism. Growth is mainly regulated by the growth axis. In mammals, the plasma concentration of growth hormone (GH) is pulsed in males and persistent in females [1,2,3]. This may be an important factor for SGD. Food intake is also a major determinant of growth, and appetite genes are crucial regulators of energy balance [4,5]. Hormones that regulate growth and metabolism affect the expression and secretion of appetite genes through the adenosine 5′-monophosphate-activated protein kinase pathway, which in turn affects energy balance [6]. Sexual differences exist in the expression and distribution of the appetite genes neuropeptide Y (NPY), neuropeptide F, and pro-opiomelanocortin (POMC) [7,8,9]. In addition, 17-β estradiol (E2) downregulates appetite-related genes and influences glucose and lipid metabolism [10]. Due to such sexual dimorphism, females exhibit higher blood glucose, enhanced lipolysis, and greater insulin sensitivity [11].

In tilapia, males grow faster than females, and the sex ratio also affects the growth phenotype [12]. Existing studies on fish growth dimorphism focused on the growth axis. In addition to the pituitary gland, the gonads of tilapia can also express GH; transplanting the ovary into muscle can promote growth [13], but growth is instead accelerated following ovariectomy [14]. Exogenous steroids can promote growth by increasing the expression of genes related to appetite or the growth axis [15]. In glucose and lipid metabolism, fish exhibit a lower tolerance to high glucose but a higher tolerance to hypoglycemia than mammals, which is attributed to insufficient peripheral glucose utilization and modulated by feeding habits [16]. The basal blood glucose of omnivorous fish is usually lower than that of carnivorous fish [17,18]. E2 injection can increase lipid droplets in the liver of male tilapia and promote lipid synthesis [19]. The mechanisms of glucose and lipid metabolism vary among fish species.

Beyond its relationship with photoperiod and circadian rhythms, melatonin also affects antioxidation, immunity, regulation of endocrines, and energy balance [20,21]. Studies on melatonin in fish have mainly focused on photoperiod and gonadal development [22,23,24]. Exogenous melatonin can inhibit the gonadal development of tilapia through immersion [25], intraperitoneal injection [26], or oral administration [27]. Melatonin can also affect growth and metabolism and decrease the specific growth rate (SGR) of tilapia [25].

The metabolites and metabolic pathways between different sexes in immature juvenile or sexually mature tilapia have not yet been examined carefully. We postulated that the metabolites differ between sexes, which leads to differences in growth in tilapia. Thus, we conducted a combined analysis of the GC-MS-based metabolome and digital gene expression (DGE) profile in the livers of immature and mature tilapia and analyzed the metabolites and pathways between males and females. Then, we used the method of intraperitoneal implantation to study the function of one of the sexually dimorphic metabolites (melatonin) in the glucose and lipid metabolism of tilapia. The study can offer abundant data support for understanding gender differences in the metabolism of tilapia and establish a reliable scientific foundation for investigating the role of melatonin in the sexual dimorphism of tilapia.

## 2. Materials and Methods

### 2.1. Fish, Diet, and Rearing Conditions

Tilapia with body weights (BWs) in the range of 5–15 g were purchased from the tilapia breeding farm of Guangdong (Panyu, Guangdong, China) and the sex ratio was close to 1:1. The fish were cultured in 180 L tanks at 27–30 °C, under a 12L:12D photoperiod. For both the sex ratio-dependent growth experiment and the post-implantation growth experiment, fish were fed once daily at 8:30 a.m. with commercial extruded feed (Tongwei Feed Co., Ltd., Foshan, China) to satiation, and the food intake of each group was recorded; for the glucose tolerance test (GTT), fish were fed quantitatively (4% body weight/day) post-implantation. Males and females were injected and sampled simultaneously to avoid errors caused by sampling sequence. After sampling, the gender of each fish was verified by histological observation of the gonads.

### 2.2. Grouping of the Sex Ratio-Dependent Growth Experiment

All tilapia were gonadally immature at the initiation of the experiment. Sex was distinguished following the Chinese national standard (SC/T 1105-2007) [28].

To explore the growth of tilapia under different sex ratios, the fish were sex-separated and randomly divided into three groups according to the following sex ratios: monosexual male group (100% male), monosexual female group (100% female), and mixed-sex group (50% male and 50% female). Each group consisted of 72 fish and was divided into triplicate subgroups. On day 0, 12 fish were sampled per group; on day 45, 24 fish were sampled per group.

### 2.3. Quantitative Analysis of Metabolic Parameters

Five biochemical parameters (glucose, triglyceride (TG), glycogen, hexokinase activity, lactate) were determined by using the corresponding commercial kits according to the manufacturer’s protocol (Nanjing Jiancheng Biochemical Institute, Nanjing, China). The catalog numbers of the kits are as follows: glucose (A154-1-1); TG (A110-1); glycogen (A043-1-1); hexokinase activity (A077-3); and lactate (A019-2-1).

### 2.4. RNA Isolation and Real-Time Quantitative PCR (qPCR) Analysis

Total RNA was extracted with a TRIzol kit manufacturer’s protocol (Invitrogen (Thermo Fisher Scientific), Carlsbad, CA, USA). The first-strand cDNA was synthesized with M-MLV reverse transcriptase (Invitrogen (Thermo Fisher Scientific), Carlsbad, CA, USA). qPCR was performed with SYBR^®^ Green real-time PCR Master Mix (Guangzhou Dongsheng Biotech Co., Ltd., Guangzhou, China), and the cycling parameters referred to previous studies [29]. For qPCR analysis of 16 relevant genes (Table S1), all primers were designed using the National Center for Biotechnology Information (NCBI) Primer-BLAST (2.17.0). Additionally, 18S rRNA and β-actin were used as reference genes.

### 2.5. GC-TOF/MS Non-Targeted Metabolomics Analysis

Livers of male and female tilapia were used for metabonomic analysis, with 10 samples per group. The gonadal developmental staging of tilapia referred to a previous report [30]. The four sample groups were defined as follows: F1—immature female (gonadal stage II); M1—immature male (gonadal stage I–II); F2—mature female (gonadal stage V); and M2—mature male (gonadal stage V).

The untargeted metabolomics profiling was performed on the XploreMET platform (Metabo-Profile Biotechnology (Shanghai) Co., Ltd., Shanghai, China). The sample preparation procedures were referred to in previously published methods with minor modifications [31]. In brief, liver tissues (50 mg each) were extracted and analyzed by GC-TOF/MS. Metabolite annotation was performed by comparing retention indices and mass spectral data with JiaLib database reference standard data via XploreMET [32], *p*-value ≤ 0.05, fold change ≥1.5 or ≤0.667, while one-way ANOVA *p*-value ≤ 0.05 was used to determine metabolites with changes in concentration. Kyoto Encyclopedia of Genes and Genomes (KEGG) pathways with sexual difference were also identified (*p* ≤ 0.05). Partial least square discriminant analysis (PLS-DA) modeling visualized differences in global metabolic profiles among groups.

### 2.6. Preparation of Melatonin Implants and Measurement of the Releasing Amount of Melatonin in the Implant

Preparation of implants: 1 mg of melatonin (MilliporeSigma (Merck KGaA), St. Louis, MO, USA) was mixed with 1 g of dimethylsiloxane solution (liquid A) and 0.1 volumes of catalyst (liquid B) (SYLGARD (Dow Corning), Midland, MI, USA) were added. We solidified it overnight and cut into strips (≈1.5 mm × 1.5 mm cross section) before use. The control group used polydimethylsiloxane implants without melatonin.

*In vitro* release rate measurement of the strips: A strip (1.5 mm × 1.5 mm × 10 mm) was placed into a 2 mL EP tube, 1 mL of PBS solution was replaced daily, followed by shaking overnight. The supernatant was collected each day, and the OD280 value was measured to calculate the release amount.

### 2.7. Intraperitoneal Implantation of Melatonin

The exogenous melatonin implant procedures used in this study are based on previous studies carried out on sea bass [33] and eel [34]. A pilot study was conducted to determine the optimal implantation dose and estimate the rate of melatonin release in the tilapia (Figure S6). Before implantation, tilapia (mean BW: 126.94 ± 2.50 g) were anesthetized and weighed. Melatonin (or control) implants (0.15 mg/g BW) were cut, loaded into sterile strip implanters, inserted via the soft skin behind the pectoral fin, and delivered into the abdominal cavity. After implantation, the wound was coated with erythromycin ointment. All fish were disinfected overnight with povidone-iodine solution. All 132 fish per group (melatonin and control) were implanted. For each group, 96 fish were allocated to the GTT, while 36 fish per group were divided into three biological replicates for the growth comparison experiment.

### 2.8. Hematoxylin and Eosin (H&E) Staining

H&E staining was performed as previously described [35]. The mesenteric adipose tissue of tilapia was fixed in formalin, dehydrated, embedded in paraffin, sectioned into 5-μm slices, and stained with an HE Staining Kit (Beyotime Biotech Inc., Shanghai, China). The adipocyte area was photographed with a microscope (Olympus Corporation, Tokyo, Japan) and measured using Image J software (version 1.50) (NIH, Bethesda, MD, USA).

### 2.9. Glucose Tolerance Test (GTT)

Ten days after implantation, tilapia were fasted for 24 h and subjected to an acute GTT, as described previously [36]. The 96 melatonin-implanted fish were separated into two groups (glucose-injected and saline-injected control). Another 96 fish implanted with control implants were injected identically. Serum was collected at time 0 (pre-injection), 1, 3, and 6 h post-injection, respectively. Male and female tilapia were mixed during GTT, with their sex recorded at sampling.

### 2.10. Statistical Analysis

Quantitative data were analyzed using SPSS (version 18.0) and expressed as mean ± SD. Pairwise comparisons were evaluated using Student’s *t*-test, while multi-group differences were assessed via one-way ANOVA with Tukey’s multiple comparisons test, with *p* < 0.05 being considered statistically significant.

The SGR and feed conversion ratio (FCR) are calculated using the following formulas:

(1) $$\mathrm{SGR}\;=\frac{\ln Average\;final\;body\;weight-\ln Average\;initial\;body\;weight}{Cultivation\;period\;(d)}\times 100\%$$

(2) $$\mathrm{FCR}=\frac{Total\;feed\;intake\;(g)}{Average\;final\;body\;weight-Average\;initial\;body\;weight}$$

## 3. Results

### 3.1. Fish Raised Monosexually Grow Faster than Those Raised in a Mixed-Sex Environment

**Figure 1:**
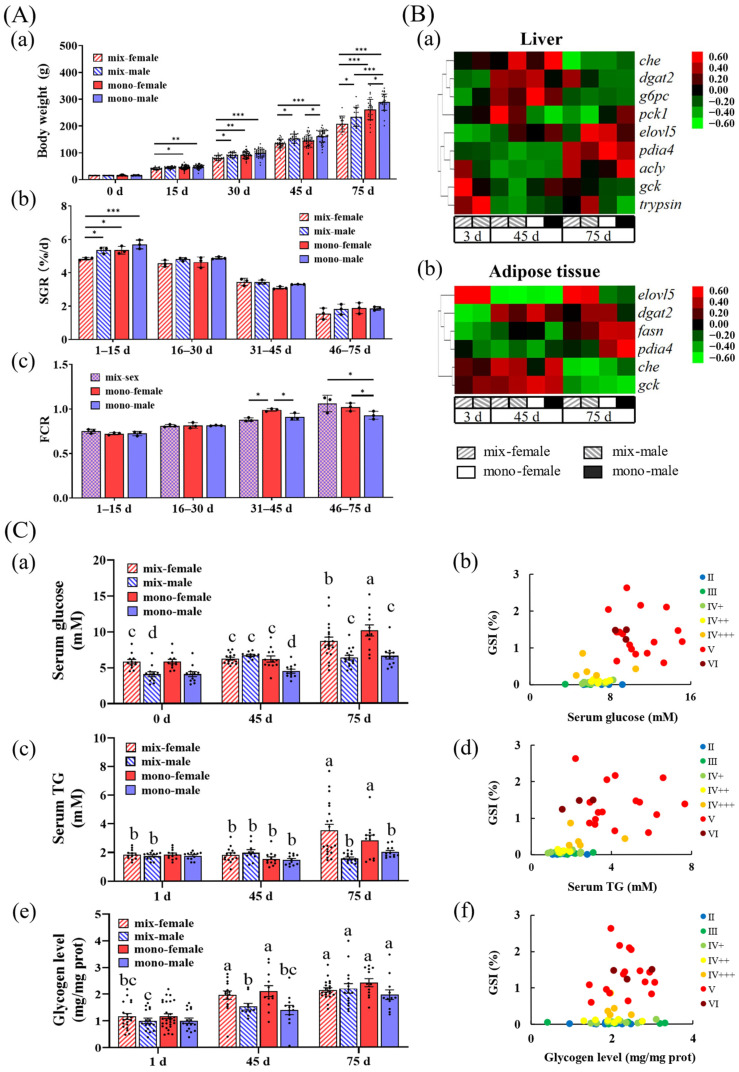
Fish raised monosexually grow faster than those raised in a mixed-sex environment. (**A**) Growth performance: (**a**) average weight (*n* = 35–45); (**b**) specific growth rate (SGR) (*n* = 3); and (**c**) feed conversion ratio (FCR) (*n* = 3). “***”, *p* < 0.001; “**”, *p* < 0.01; and “*”, *p* < 0.05. (**B**) qPCR heatmap: Relative mRNA expression of genes in the (**a**) liver and (**b**) adipose tissue. Red indicates relatively higher mRNA expression, while green indicates relatively lower expression. The data were normalized to the mean and clustered by gene. (**C**) Metabolic parameter levels: (**a**) serum glucose level (*n* = 12–16); (**c**) serum triglyceride (TG) level (*n* = 12–16); and (**e**) liver glycogen level (*n* = 14–20). Different letters indicate significant groups at each identical time point, *p* < 0.05. Relationship between ovarian developmental stage, female GSI, and the level of the following: (**b**) serum glucose; (**d**) TG; and (**f**) hepatic glycogen. Roman numerals (II, III, IV, V, VI) represent the staging of ovarian development.

The average weight of monosexual group was greater than that of the mixed-sex group, and the average weight of males was higher than that of females (Figure 1(Aa)). The SGR of mixed-sex females during days 0–15 and monosexual females during days 31–45 was lower than other groups (Figure 1(Ab)). In the first 30 days, there was no significant difference in the FCR among all groups. During days 31–45, the FCR of monosexual females was higher than that of the other groups, and during days 46–75, the mixed-sex group had a higher FCR than that of the monosexual male (Figure 1(Ac)). 

Relative mRNA expression of genes in the growth axis showed that the level of *gh* mRNA in the pituitary of the F1 (immature female) group was higher than that in the M1 (immature male) and F2 (mature female) groups (Figure S1(a1)); the levels of growth hormone receptor (*ghr1* and *ghr2*) mRNA in the liver of F2 and M2 (mature male) were significantly higher, and there were no sexual differences (Figure S1(b2)/(b3)). The levels of insulin-like growth factor 1 (*igf1*) in F2 and M2 were higher, and M2 was higher than F2 (Figure S1(b1)). The level of *gh* and *ghr1* mRNA in the gonads of males were higher than that of females (Figure S1(c1)/(c2)). There was no difference in the *ghr2* mRNA level in the gonad (Figure S1(c3)).

During days 0–45, the food intake increased; after day 45, the food intake fluctuated greatly. In general, the food intake of monosexual males was significantly higher than that of the mixed-sex group. During days 61–75, the food intake of monosexual females was significantly higher than the mixed-sex group (Figure S2a). The mRNA levels of appetite-related genes, *npy* and agouti-related protein (*agrp*) in the hypothalamus of immature females were higher than those in other groups (Figure S2(b1)/(b2)).

The results of the qPCR of relevant genes in the liver and adipose tissue showed that the levels of glucokinase (*gck*), cholinesterase (*che*), and trypsin mRNA were higher in immature females, and the levels of gluconeogenesis-related (glucose-6-phosphatase, *g6pc*) and lipid metabolism-related (diacylglycerol Acyltransferase 2, *dgat2*; fatty acid synthase, *fasn*; elongase of very long chain fatty acids 5, *elovl5*) mRNA were higher in sexually mature groups (Figure 1(Ba)/(Bb) and Figure S3).

Serum glucose, serum TG, and liver glycogen increased gradually as fish grew up (Figure 1(Ca)/(Cc)/(Ce)). Females with more mature ovarian development tended to have higher serum glucose and TG (Figure 1(Cb)/(Cd)). The basal serum glucose of females was higher than that of males (Figure 1(Ca)). The serum TG of females increased significantly after sexual maturity (Figure 1(Cc)). There was no significant difference in liver glycogen levels after sexual maturity (Figure 1(Cf)).

### 3.2. Melatonin in the Liver of Mature Female Tilapia Was Significantly Higher than Those of Mature Male

**Figure 2:**
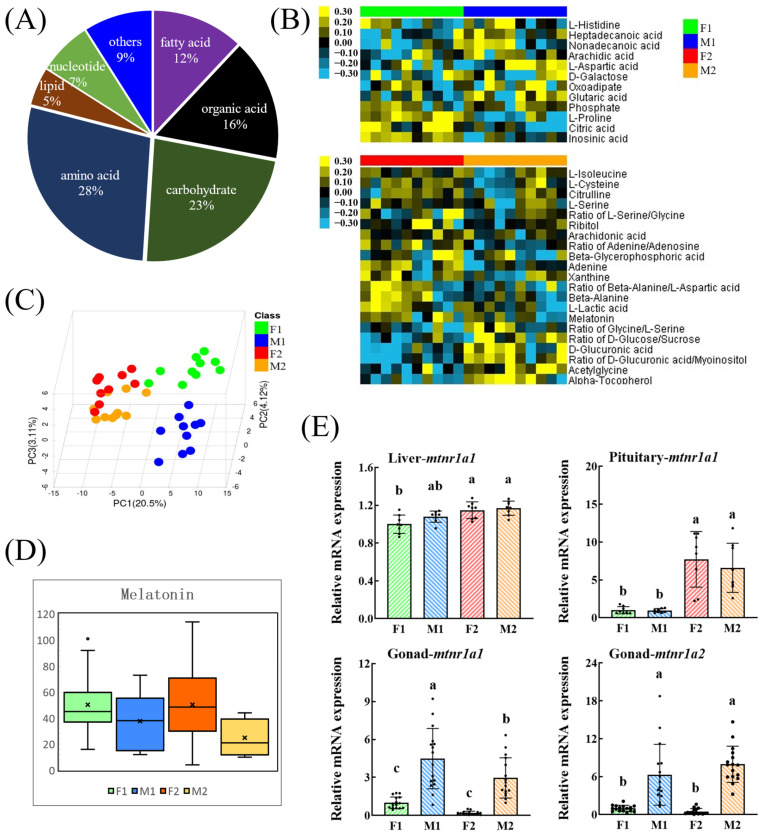
Metabolomics analysis of liver samples from male and female tilapia. (F1, immature female; M1, immature male; F2, mature female; M2, mature male). (**A**) Metabolite classes and compositions. (**B**) Sex-differential metabolites. Yellow: higher concentration; blue: lower concentration (*p* < 0.1). (**C**) PLS-DA clustering. (**D**) Boxplot of melatonin level. “×”, mean of the data. (**E**) Relative mRNA expression of *mtnr1a1* and *mtnr1a2* in the pituitary (*n* = 8), liver (*n* = 8), and gonad (*n* = 16). Different letters indicate significant differences among groups (*p* < 0.05).

A total of 230 peaks were detected, 122 metabolites were annotated, and the ratios of 29 metabolite pairs were analyzed (Figure 2A). The data of these four groups can be well separated by PLS-DA clustering (Figure 2C). Z-score analysis identified more differential metabolites between the F1/M1 and F2/M2 groups (Figure S4).

Several metabolite levels were different between male and female: 12 metabolites (or their ratios) were different between F1 and M1 and 21 metabolites (or their ratios) were different between F2 and M2 (Figure 2B, Table 1). Among them, the concentration of melatonin in the liver of mature females (F2) was significantly higher than that of males (M2) (Figure 2D and Table 1). The mRNA levels of melatonin receptors *mtnr1a1* and *mtnr1a2* in the gonad of females were significantly higher than that in males. No sexual difference was observed in *mtnr1a1* mRNA levels in the liver or pituitary (Figure 2E).

### 3.3. The Multi-Omics Approach Showed Sexual Differences in Several Metabolic Pathways

KEGG pathways identified by either metabolomics (Table S2) or DGE (Table S3) indicated that males exhibited more active adipogenesis and gluconeogenesis, while females showed greater activity in the estrogen signaling pathway and glucose catabolism. This indicates that males can store more energy, whereas females require more energy expenditure (Figure S5).

**Figure 3:**
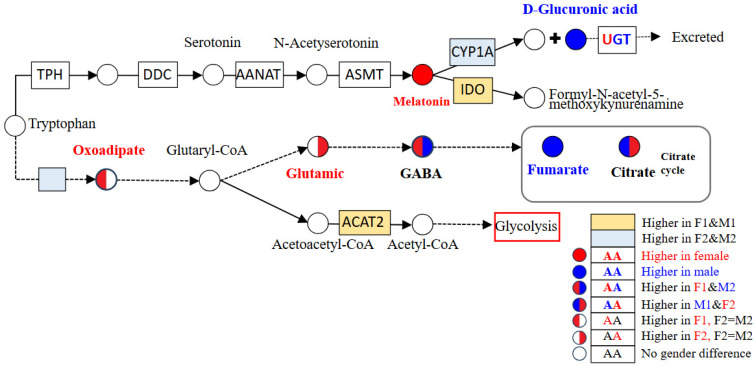
Sexual dimorphism in the tryptophan metabolism pathway. Circles represent the metabolites in the pathway: red, higher concentration in females; blue, higher concentration in males; white, no sexual difference. The color of the left semicircle in the circle indicates the difference between F1 and M1, while the right semicircle indicates the difference between F2 and M2.

Among the metabolites with sexual differences, the levels of 2-oxoadipate, melatonin, and D-glucuronic acid, which are associated with the tryptophan metabolic pathway, were consistent with the differences in related metabolic enzymes identified by DGE (Table 1 and Figure 3). Therefore, we investigated the effects of melatonin on the metabolism and growth of tilapia.

### 3.4. Melatonin Implantation Inhibited the Food Intake and Growth and Affected the Glucose and Lipid Metabolism of Tilapia

**Figure 4:**
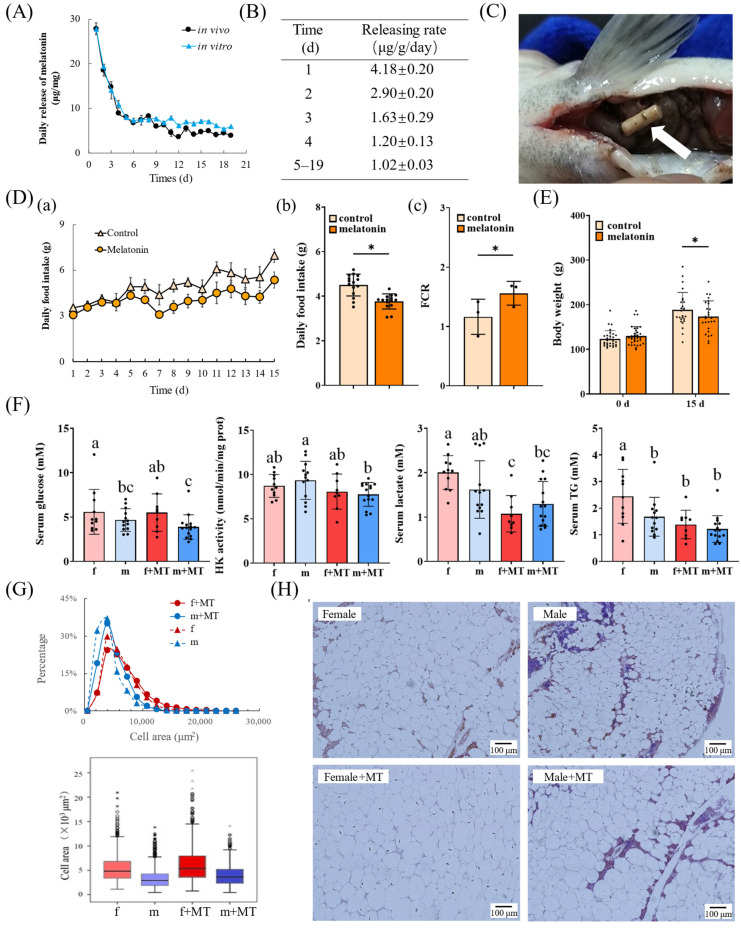
Effects of melatonin implantation on the food intake, growth, and metabolism of tilapia. MT, melatonin implant group. (**A**) *In vitro* and *in vivo* release curves of melatonin implants (*n* = 3). (**B**) *In vivo* release rate of melatonin implants (*n* = 3). (**C**) White arrow indicates the implant in the abdominal cavity. (**D**) Daily food intake (**a**,**b**) and FCR (**c**) within 15 days post-implantation (*n* = 3). “*”, *p* < 0.05. (**E**) Average weight (*n* = 30). “*”, *p* < 0.05. (**F**) Effects of implantation of melatonin on the serum glucose, TG, lactate, and liver hexokinase activity of tilapia (*n* = 9–15); different letters indicate significant differences among groups. (*p* < 0.05). f, female; m, male. (**G**) Statistics of adipocyte area in sections. Each group contained statistics for more than 1000 cells. “●”, outliers (values between 1.5 × interquartile range (IQR) and 3 × IQR from the upper quartiles); “*”, extreme outliers (values beyond 3 × IQR from the upper quartiles). (**H**) HE staining of adipose tissue in each group, scale bar = 100 μm.

We measured the daily melatonin release from unused melatonin implants and the *in vitro* daily melatonin release from implants retrieved after *in vivo* implantation (Figure 4A,B). From day 1 to day 4, the total daily melatonin release per mg of the strip was 27.84, 18.53, 14.77, and 8.92 μg, respectively. After day 5, the daily release *in vivo* was relatively stable (Figure 4B). Preliminary experiments confirmed that the melatonin implantation dosage is within the safe range (Figure S6). The intraperitoneal implantation site is shown in Figure 4C.

After implantation, food intake decreased, weight gain slowed down, and the FCR increased (Figure 4D,E). The basal serum glucose of males was significantly lower than that of females, and melatonin could slightly reduce the basal serum glucose of both male and female tilapia (no significant difference). In addition, serum lactic acid and TG levels of female fish decreased significantly; hepatic hexokinase activity of males decreased, while that of females remained unchanged (Figure 4F). We analyzed changes in mesenteric adipocyte size in tilapia 15 days after implantation. The median adipocyte size in female tilapia was larger than that in males, and melatonin implantation tended to increase adipocyte size (no significant difference) (Figure 4G,H).

### 3.5. Melatonin Implantation Improved the Glucose Tolerance of Tilapia, Especially in Females

**Figure 5:**
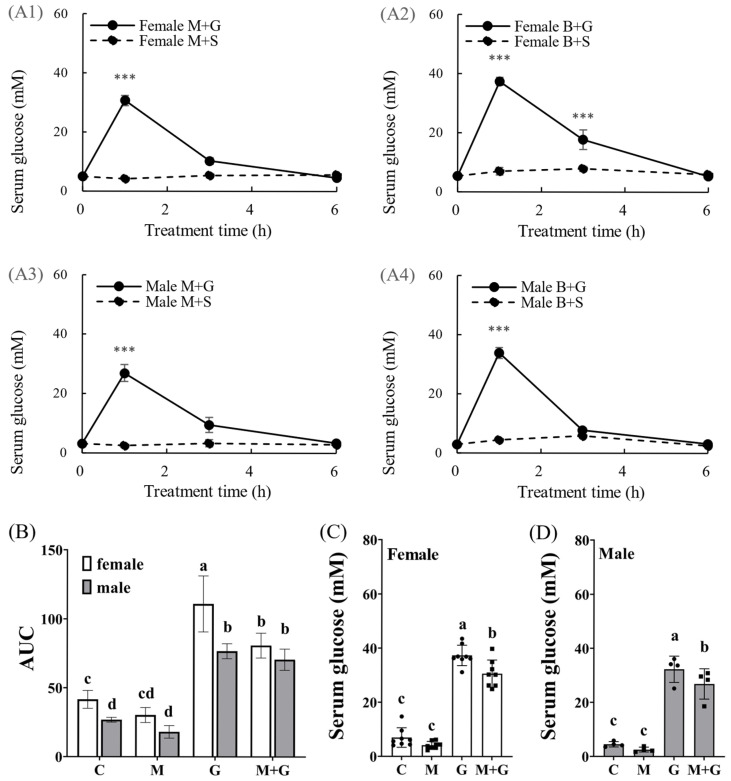
Effect of melatonin implantation on glucose tolerance in tilapia. (**A1**–**A4**) M: melatonin implantation, B: blank strip implant, G: 1 mg/g glucose injection, S: PBS control injection (*n* = 4–8), and “***”, *p* < 0.01. (**B**) Area under the curve (AUC), C, blank + PBS; M, melatonin + PBS; G, blank + glucose; M + G, melatonin + glucose. (**C**) Serum glucose concentrations of female tilapia at 1 h after intraperitoneal injection (*n* = 8), (**D**) serum glucose concentrations of male tilapia at 1 h after intraperitoneal injection (*n* = 4). Different letters indicate significant differences among groups. (*p* < 0.05).

Serum glucose was detected at 0, 1, 3, and 6 h after glucose or saline injection. Glucose treatment increased serum glucose levels at 1 h, with normalization by 6 h (Figure 5A1–A4). At 3 h, serum glucose nearly returned to normal, except in females with control implants (Figure 5A2). Post-treatment, the area under the curve (AUC) of females with control implants was significantly higher than in other groups (Figure 5B). At 1 h post-glucose injection, melatonin-implanted tilapia had significantly lower serum glucose levels than control implant groups (Figure 5C,D).

## 4. Discussion

In the growth experiment, females in the monosexual group exhibited a growth rate that was comparable to males in the mixed-sex group. No female spawning was observed, indicating that spawning did not contribute to the observed growth differences. Monosexual male tilapia exhibit faster growth, lower FCR, and androgen-mediated growth promotion [12]. Tilapia communicate via steroid hormones in urine detecting olfactory glucuronic acid-conjugated environmental steroids [37], which facilitate female reproduction [38,39,40]. Exogenous E2 downregulates hepatic *ghr1* and *ghr2* expression, whereas testosterone only reduces the *ghr2* levels, indicating sex-specific regulation of GHRs [41]. Territorial males exhibit higher serum 11-ketotestosterone concentrations, suggesting growth modulation by pheromones [42].

The gonads of tilapia can directly regulate growth by secreting GH [13]. We detected significantly higher *gh* and *ghr1* mRNA levels in testes than in ovaries, which may partially explain the growth differences. GHR1 functions vary among fish species and in some species, it may be the receptor for somatolactin [43,44]. The expression patterns of growth axis genes in tilapia vary with different gonadal developmental stages and differ across tissues [45,46]; thus, further research should be conducted in conjunction with developmental status.

DGE and qPCR identified sexually dimorphic genes in the liver. Glycolytic genes are upregulated in immature females, while gluconeogenic and lipid synthesis genes are upregulated in sexually mature groups, indicating that energy metabolism may shift from consumption to storage with individual development. Combined with serum glucose, TG, and liver glycogen data, females exhibit enhanced anabolism during gonadal development. E2 can affect glucose metabolism and energy balance [47]. Elevated serum TG in gonad stage V females may be linked to a sharp increase in E2, as E2 increases TG via cholesterol ester transfer protein [48] and lipid deposition-associated genes [19]. Thus, the differences in glucose and lipid metabolism between male and female tilapia are related to the E2 and estrogen receptor α signal pathways, and the differences in metabolism may eventually lead to growth differences between male and female fish.

Metabolomic analysis identified sexually dimorphic metabolites involved in glucose and lipid metabolic pathways in the liver, including D-galactose, L-lactic acid, citric acid, glucuronic acid, and arachidonic acid. Among them, citric acid is an important metabolite of tricarboxylic acid cycle. Glucuronic acid supports steroid metabolism and sex pheromone perception [37], and arachidonic acid is a downstream metabolic product of lecithin and participates in the metabolism of TG, with its sexual dimorphism being consistent with the findings in the liver of humans [49]. Sexually mature tilapia store energy as lipids or glycogen, with males being more active. For sexual dimorphism, males have more robust long-chain fatty acid synthesis and enriched vitamin D3 metabolism, while females show greater glycolytic activity, enhanced cholesterol synthesis, and more active progesterone and corticosteroid production. Researchers have analyzed sexual dimorphisms in the brain of tilapia, and elevated E2 levels in females upregulate *pomc* expression and inhibit feeding behavior in female tilapia [50]. In tilapia muscle transcriptomes, cell growth- and proliferation-related pathways are upregulated in males, while cell division regulatory pathways are upregulated in females [51]. Growth dimorphism may be partially attributed to sexually dimorphic metabolism in the liver; these findings are consistent with transcriptomic studies in other fish species [52,53,54].

The tryptophan metabolic pathway is one of the common differential metabolic pathways identified by metabolomics and DGE in this study. Exogenous melatonin could make the feeding behavior, growth phenotype, and adipocyte area of male tilapia closer to those of females. The glucose tolerance of female tilapia was significantly higher than that of males. Melatonin signaling is a key regulator of glucose homeostasis and energy metabolism [55]. In rat models, melatonin can affect lipid metabolic pathways, thereby reducing obesity [56,57,58]. Our results regarding exogenous melatonin in tilapia were consistent with these studies.

## 5. Conclusions

Our study indicates that growth dimorphism in tilapia is closely associated with sexual differences in hepatic glucose, lipid, and tryptophan metabolism, and is influenced by gonadal development levels, while exogenous melatonin can regulate tilapia growth by affecting glucose and lipid metabolic pathways. Future research should cover all gonadal developmental stages to capture undiscovered early sexual differences in metabolism; further in-depth studies on melatonin receptors are also needed to clarify the specific pathways through which melatonin regulates metabolism.

## Figures and Tables

**Table 1:** Differential metabolites between males and females.

(a) M1 vs. F1						
Class	Name	HMDBID	KeggID	*p* (*t*-Test)	FC	*p* (FDR)
Amino Acid	L-Proline	HMDB00162	C00148	2.01 × 10^−2^	0.7	2.02 × 10^−2^
	L-Aspartic acid	HMDB00191	C00049	3.50 × 10^−2^	1.4	3.57 × 10^−2^
Carbohydrates	D-Galactose	HMDB00143	C00984	0.068	1.7	0.071
Fatty Acids	Nonadecanoic acid	HMDB00772	C16535	4.21 × 10^−2^	1.4	4.35 × 10^−2^
	Heptadecanoic acid	HMDB02259	NA	0.073	1.5	0.077
Nucleotide	Inosinic acid	HMDB00175	C00130	0.075	0.8	0.080
Organic Acids	Glutaric acid	HMDB00661	C00489	3.61 × 10^−3^	1.8	3.61 × 10^−3^
	2-Oxoadipate	HMDB00225	C00322	3.72 × 10^−2^	1.2	3.82 × 10^−2^
	Citric acid	HMDB00094	C00158	4.89 × 10^−2^	0.7	0.051
Phosphate	Phosphate	HMDB01429	C00009	2.19 × 10^−2^	1.1	2.22 × 10^−2^
**(b) M2 vs. F2**						
**Class**	**Name**	**HMDBID**	**KeggID**	***p* (*t*-Test)**	**FC**	***p* (FDR)**
Amino Acid	Beta-Alanine	HMDB00056	C00099	1.28 × 10^−2^	0.4	1.31 × 10^−2^
	L-Cysteine	HMDB00574	NA	2.22 × 10^−2^	0.5	2.31 × 10^−2^
	Citrulline	HMDB00904	C00327	0.050	0.7	0.054
	Acetylglycine	HMDB00532	NA	0.079	1.2	0.088
	L-Serine	HMDB00187	C00065	0.080	0.8	0.089
Carbohydrates	D-Glucuronic acid	HMDB00127	C00191	4.39 × 10^−2^	1.6	4.67 × 10^−2^
Fatty Acids	Arachidonic acid	HMDB01043	C00219	0.067	0.7	0.074
Indoles	Melatonin	HMDB01389	C01598	3.97 × 10^−2^	0.4	4.19 × 10^−2^
Lipids	Beta-Glycerophosphoric acid	HMDB02520	C02979	0.085	0.6	0.097
Nucleotide	Adenine	HMDB00034	C00147	2.63 × 10^−3^	0.7	2.63 × 10^−3^
	Xanthine	HMDB00292	C00385	0.062	0.5	0.068
Organic Acids	L-Lactic acid	HMDB00190	C00186	7.26 × 10^−3^	0.5	7.36 × 10^−3^
Vitamin	Alpha-Tocopherol	HMDB01893	C02477	1.54 × 10^−2^	1.9	1.59 × 10^−2^

HMDBID: The unique identifier of metabolites in the Human Metabolome Database (HMDB); FC (fold change): the ratio of metabolite content between two groups, calculated as “M1/F1 or M2/F2”; FDR: false discovery rate-corrected *p*-value by Benjamini–Hochberg method. *p* ≤ 0.05, FC < 1, significantly decreased in males; *p* ≤ 0.05, FC > 1, significantly increased in males; 0.05 < *p* ≤ 0.1, FC < 1, decreased in males; and 0.05 < *p* ≤ 0.1, FC > 1, increased in males.

## Data Availability

The original contributions presented in this study are included in the article. Further inquiries can be directed to the corresponding author.

## Supplementary Materials

The following supporting information can be downloaded at: https://www.mdpi.com/article/10.3390/biom16010015/s1. Table S1: Primers used in this study; Table S2: Differential KEGG pathway identified by metabonomic; Table S3: Differential KEGG pathways in the liver of tilapia identified by DGE; Figure S1: Relative mRNA expression of genes in the growth axis; Figure S2: Average daily feed intake and appetite gene expression; Figure S3: Relative mRNA expression of genes in the liver and adipose tissue; Figure S4: Z-score plot depicting the overall metabolite variations among groups; Figure S5: Integrated analysis of DGE and metabolomic profiles in glucose and lipid metabolic pathways; Figure S6: Intraperitoneal injection of melatonin at different concentrations.
