# Supplementary material for: The Involvement of Melatonin in the Dimorphism of Glucose and Lipid Metabolism of Tilapia
Source: Biomolecules. 2025 Dec 21;16(1):15. doi: 10.3390/biom16010015 (PMC12838915; doi:10.3390/biom16010015)
Supplement: Supplementary file 1 [file biomolecules-16-00015-s001.zip › Figure S1.pdf]

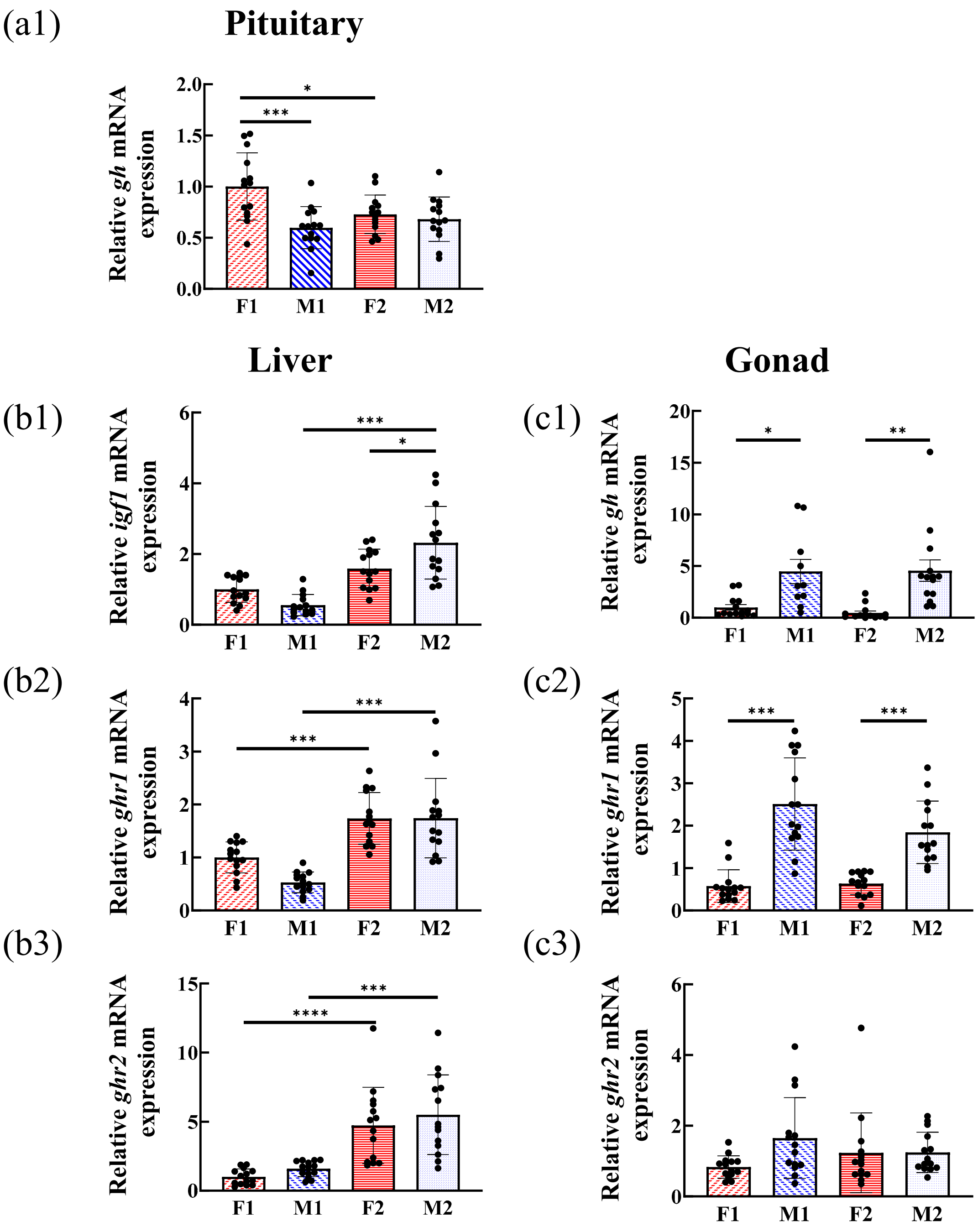


**Figure S1. Relative mRNA expression of genes in the growth axis.** (**a1**) gh mRNA level in the pituitary, (**b1-b3**) igf1/ghr1/ghr2 mRNA level in the liver, (**c1-c3**) gh/ghr1/ghr2 mRNA level in the gonad. (F1: Immature female, M1: Immature male, F2: mature female, M2: mature male). (n=15) (C) (a) Average daily food intake (n=15), (b1-2) npy and agrp mRNA level in the hypothalamus (n=15). *** indicates $p < 0.001$, ** indicates $p < 0.01$, * indicates $p < 0.05$.
