# Supplementary material for: The Involvement of Melatonin in the Dimorphism of Glucose and Lipid Metabolism of Tilapia
Source: Biomolecules. 2025 Dec 21;16(1):15. doi: 10.3390/biom16010015 (PMC12838915; doi:10.3390/biom16010015)
Supplement: Supplementary file 1 [file biomolecules-16-00015-s001.zip › Figure S2.pdf]

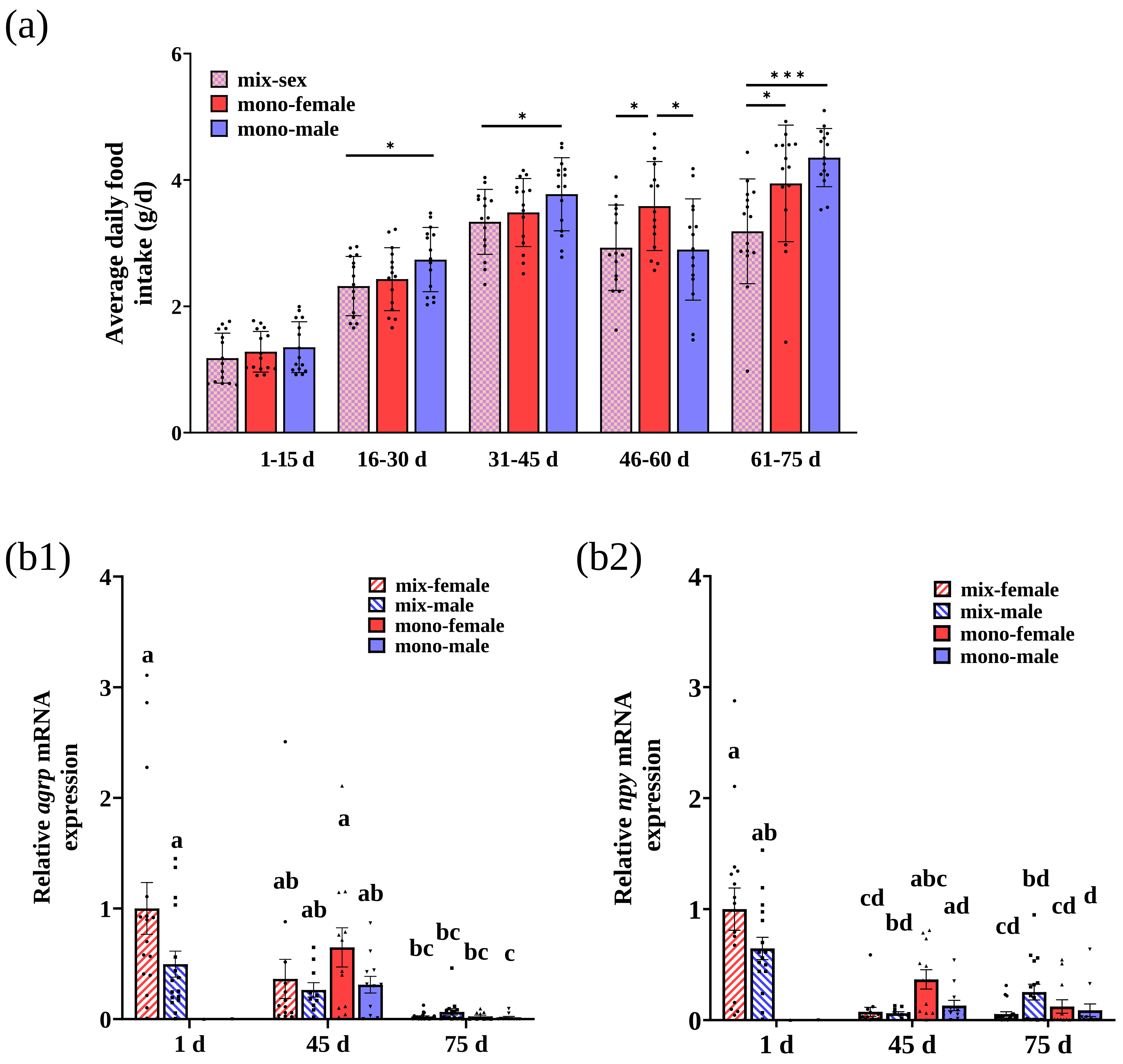

Figure S2. Average daily feed intake and appetite gene expression. (**a**) Average daily food intake (n=15), *** indicates p < 0.001, * indicates p < 0.05. (**b1**,**b2**) *npy* and *agrp* mRNA level in the hypothalamus (n=15), different letters indicate significant differences among groups at each identical time point. (p < 0.05).
