# Supplementary material for: The Involvement of Melatonin in the Dimorphism of Glucose and Lipid Metabolism of Tilapia
Source: Biomolecules. 2025 Dec 21;16(1):15. doi: 10.3390/biom16010015 (PMC12838915; doi:10.3390/biom16010015)
Supplement: Supplementary file 1 [file biomolecules-16-00015-s001.zip › Figure S3.pdf]

(a) Liver

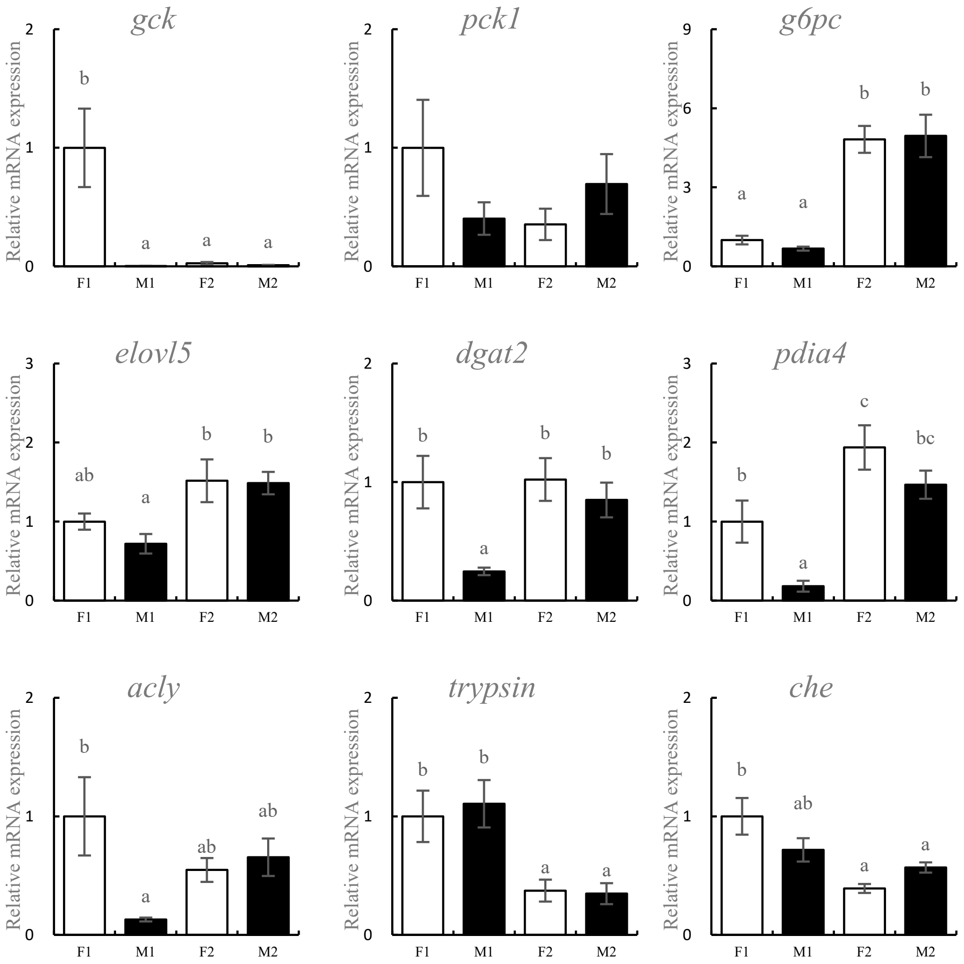

(b) Adipose tissue

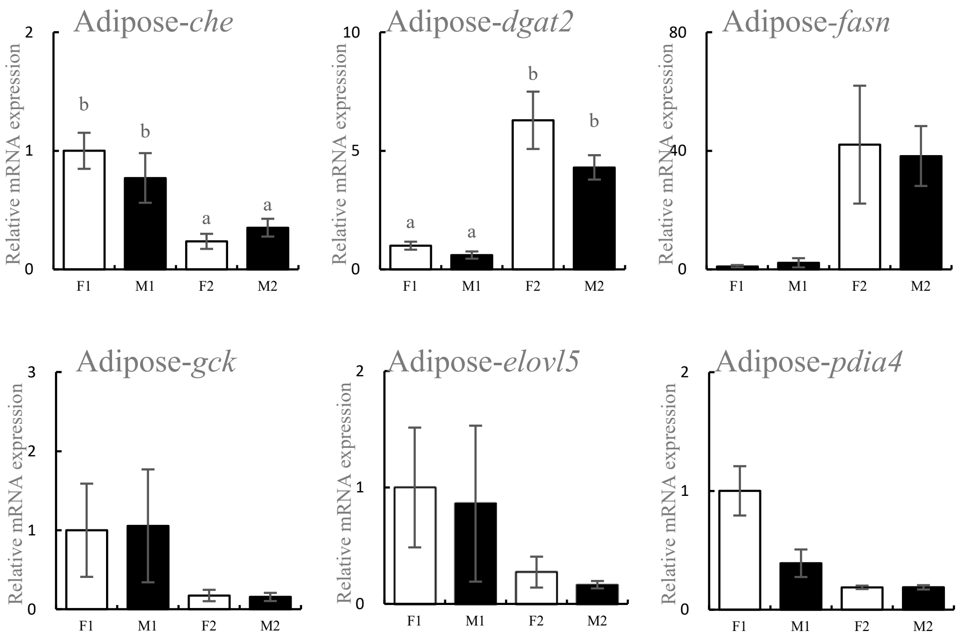

Figure S3. Relative mRNA expression of genes in the liver and adipose tissue. (F1: Immature female, M1: Immature male, F2: mature female, M2: mature male) (n=15). different letters indicate significant differences among groups at each identical time point. ($p < 0.05$).
