# Supplementary material for: The Involvement of Melatonin in the Dimorphism of Glucose and Lipid Metabolism of Tilapia
Source: Biomolecules. 2025 Dec 21;16(1):15. doi: 10.3390/biom16010015 (PMC12838915; doi:10.3390/biom16010015)
Supplement: Supplementary file 1 [file biomolecules-16-00015-s001.zip › Figure S6.pdf]

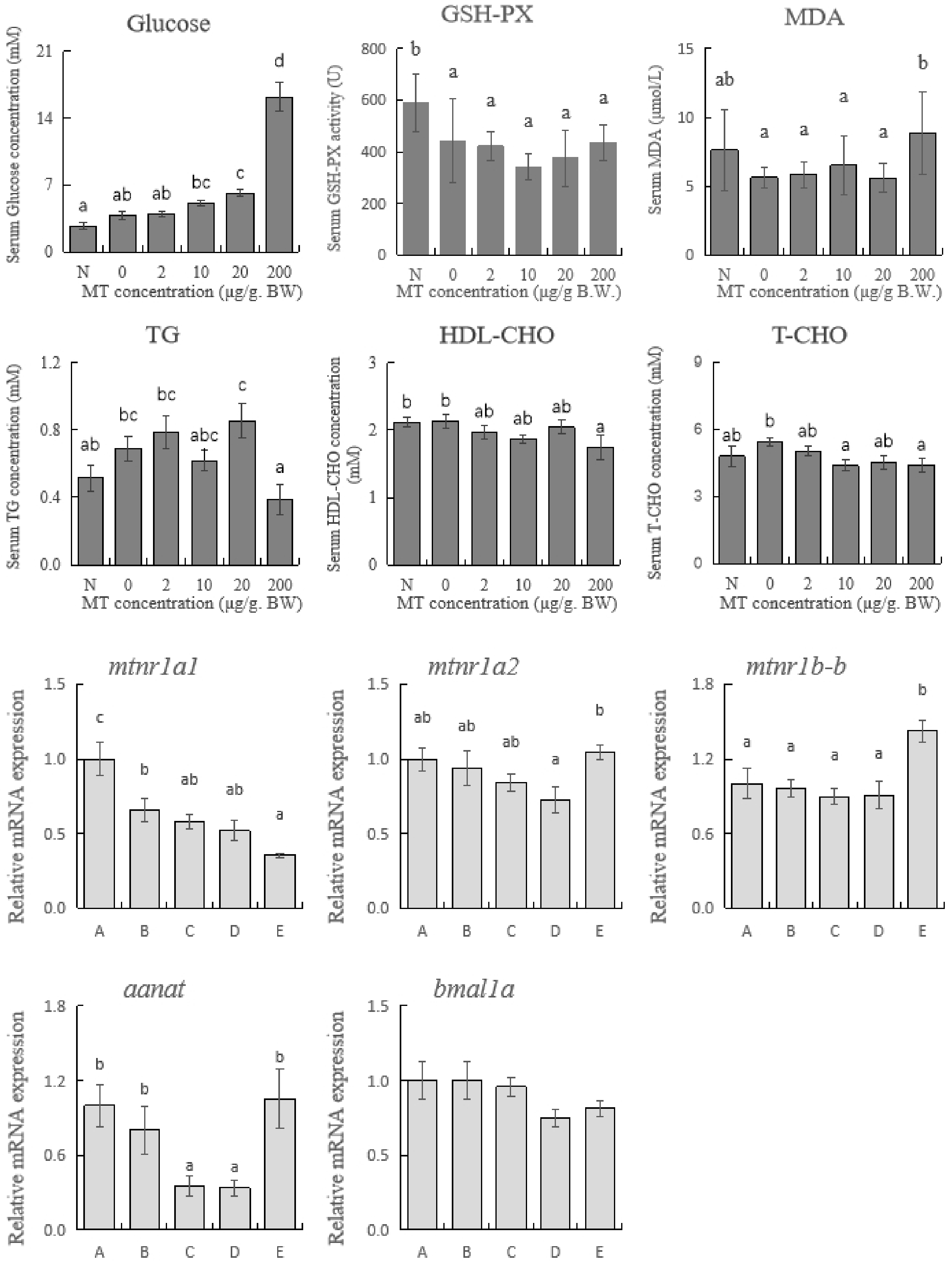

Figure S6. Intraperitoneal injection of melatonin at different concentrations:
(a) Effects of gradient MT injection on the concentrations of serum metabolites in tilapia. Data are presented as mean ± standard error (n=8), and multiple comparisons among groups were performed using Duncan's method. Letter N represents the control group with needle pricking only but no injection.
(b) Effects of gradient MT injection on melatonin receptor, aanat, and bmal1a mRNA levels in the liver of tilapia. Data are presented as mean ± standard error (n=8), and multiple comparisons among groups were performed using Duncan's method. Letters A/B/C/D/E correspond to MT injection concentrations of 0/2/10/20/200 μg/g, respectively.
