# Supplementary material for: The Involvement of Melatonin in the Dimorphism of Glucose and Lipid Metabolism of Tilapia
Source: Biomolecules. 2025 Dec 21;16(1):15. doi: 10.3390/biom16010015 (PMC12838915; doi:10.3390/biom16010015)
Supplement: Supplementary file 1 [file biomolecules-16-00015-s001.zip › Table S1.pdf]

Table S1. Primers used in this study.

Name	Sequences (5`—3`)	Accession No.
NPY-F	ACAAGACAGAGGTATGGGAAGA	XM_003448854.5
NPY-R	CAATGTGGTGATGCTGCC	
AgRP-F	ACACGTCTGCAGCCTTACAG	XM_005447762.3
AgRP-R	GTGTCACAGGGGTCACAACA	
GH-F	ACAGCCAGCGTTTGTTCTCCATTG	MH936528.1
GH-R	GGAAACTCCCAGGACTCAACCAGT	
IGF I-F	TGCGATGTGCTGTATCTCCTG	XM_019346352.2
IGF I-R	GCCATAGCCTGTTGGTTTATTG	
GHR1-F	CAAGTCCTTCCGGGCTAA	AY973232.1
GHR1-R	ACTGTCGCTGAATGTCCAAT	
GHR2-F	CAGCACCGAGACAACAGC	AY973233.1
GHR2-R	TCAGGATGCCCACTAAAC	
DGAT2-F	GGGACGGGAAATGCT	XM_003458972.5
DGAT2-R	GTTCTCGCCGAAGGAA	
ACLY-F	GGAGGTTGCCGAGGTA	XM_003442027.5
ACLY-R	AAGGCCAGAGGTGAGG	
PDIA4-F	CATAGAGGGCAAAGACACG	XM_003443497.5
PDIA4-R	GCCACAGGTATAGGAGGGT	
CHE-F	CACTCATCAATCAATCCCTG	XM_003441619.5
CHE-R	GGTCATATTAACTTCCTCCTTT	
ELOVL5-F	GAGATCAGCGGGTTCG	NM_001279460.1
ELOVL5-R	TTGTAGCCTCCGTGCC	
trypsin-F	AGTCCCAATCCTGTCTGA	XM_003458790.5
trypsin-R	TGTATCGGGAAATAGTGTC	
GCK-F	ACAGAGTGGTGGACGAAG	XM_003451020.5
GCK-R	CACCTGTGAGACATAGCG	
PCK1-F	CCAGACCGCCGACAAATTATC	XM_003448375.3
PCK1-R	GTTGGTGATGCCCAGGATCA	
FASN-F	CCAGACTTCAGAGACTCCATTC	XM_003454056.5
FASN-R	TGCGTGAACTGTGTCTTCAA	
G6PC-F	CGCCAGCATGAAGAAGTATTTC	XM_013273429.2
G6PC-R	CACCACTTCTGGGCTTTCTC	
β-actin-F	ACCTTCTACAACGAGCTGAGAG	XM_003443127.5
β-actin-R	GCCTGGATGGCAACGTACA	
18S-F	AGAAACGGCTACCACATCC	XR_003216134.1
18S-R	CACCAGACTTGCCCTCCA	
