# Supplementary material for: The Involvement of Melatonin in the Dimorphism of Glucose and Lipid Metabolism of Tilapia
Source: Biomolecules. 2025 Dec 21;16(1):15. doi: 10.3390/biom16010015 (PMC12838915; doi:10.3390/biom16010015)
Supplement: Supplementary file 1 [file biomolecules-16-00015-s001.zip › Table S2.pdf]

Table S2. Differential KEGG pathway identified by metabonomics.

(a) F1 vs M1

Pathway_name	Up (KeggID)	Down (KeggID)
Pantothenate and CoA biosynthesis*	C00049; C00106	C00097; C00183
beta-Alanine metabolism*	C00135; C00049; C00106	C00315
Arginine biosynthesis*	C00122; C00049; C00077	
Biosynthesis of unsaturated fatty acids*	C01595; C06425	C00712; C01530
Alanine, aspartate and glutamate metabolism*	C00122; C00049	C00158; C00334
Valine, leucine and isoleucine biosynthesis*		C00188; C00183
Arginine and proline metabolism*	C00077	C00334; C00148; C00315
Galactose metabolism	C00984	C00159; C00794
Glutathione metabolism	C00077	C00097; C00315
Glyoxylate and dicarboxylate metabolism	C00160	C00158; C00065
Glycine, serine and threonine metabolism		C00188; C00065; C00097
Histidine metabolism	C00135; C00049	

(b) F2 vs M2

Pathway_name	Up (KeggID)	Down (KeggID)
Valine, leucine and isoleucine biosynthesis*		C00188; C00407; C00123
Alanine, aspartate and glutamate metabolism*	C00158; C00334	C00152; C00025
Ascorbate and aldarate metabolism*	C00191	C00137
Galactose metabolism	C00984	C00137; C00794
Arginine biosynthesis		C00327; C00025
Glyoxylate and dicarboxylate metabolism	C00158	C00065; C00025
Butanoate metabolism	C00334	C00025
Tryptophan metabolism	C00322	C01598;

Annotation: $p < 0.1$, '*' indicates $p < 0.05$. 'up' indicates the concentration of the metabolite is higher in males, 'down' indicates the concentration of the metabolite is higher in females.
