# Supplementary material for: The Involvement of Melatonin in the Dimorphism of Glucose and Lipid Metabolism of Tilapia
Source: Biomolecules. 2025 Dec 21;16(1):15. doi: 10.3390/biom16010015 (PMC12838915; doi:10.3390/biom16010015)
Supplement: Supplementary file 1 [file biomolecules-16-00015-s001.zip › Table S3.pdf]

Table S3. Differential KEGG pathways in the liver of tilapia identified by digital DGE.

Pathway name（F1 vs M1）	ID	Samples	Background
Steroid biosynthesis	ko00100	7	82
Terpenoid backbone biosynthesis	ko00900	4	103
Glycerolipid metabolism	ko00561	2	117
Glycolysis / Gluconeogenesis	ko00010	2	132
Synthesis and degradation of ketone bodies	ko00072	1	15
Starch and sucrose metabolism	ko00500	2	147
Protein processing in endoplasmic reticulum	ko04141	5	147
Thyroid hormone synthesis	ko04918	3	74
Metabolic pathways	ko01100	17	4675
Biosynthesis of secondary metabolites	ko01110	10	2153
**Pathway name（F2 vs M2）**	**ID**	**Samples**	**Background**
PPAR signaling pathway	ko03320	4	67
Cholesterol metabolism	ko04979	4	60
Glycerophospholipid metabolism	ko00564	4	162
Arachidonic acid metabolism	ko00590	3	121
Steroid hormone biosynthesis	ko00140	3	147
Cysteine and methionine metabolism	ko00270	6	163
Glycine, serine and threonine metabolism	ko00260	3	146
Tyrosine metabolism*	ko00350	3	150
Glutathione metabolism	ko00480	2	82

* Pathways screened from significantly enriched KEGG pathways, $P < 0.05$, focusing on those related to glucose metabolism and lipid metabolism.
