# Supplementary material for: The Involvement of Melatonin in the Dimorphism of Glucose and Lipid Metabolism of Tilapia
Source: Biomolecules. 2025 Dec 21;16(1):15. doi: 10.3390/biom16010015 (PMC12838915; doi:10.3390/biom16010015)
Supplement: Supplementary file 1 [file Figure S4.pdf › Figure S4.pdf]

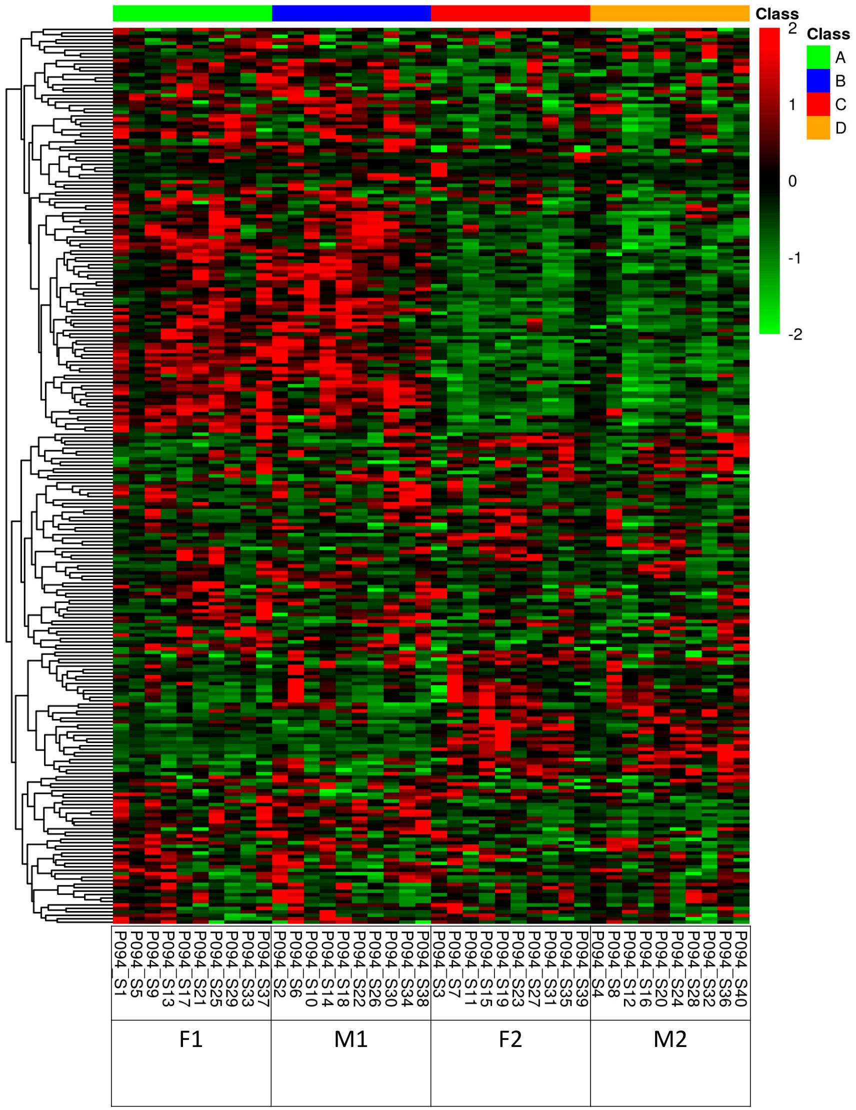


**Figure S4 Z-score plot depicting the overall metabolite variations among groups.** red color indicates relative higher concentration while green means relative lower concentration levels. F1: immature male; M1:immature male; F2: mature female; M2: mature male. Each column represents one sample, with 10 samples per group.
