# Supplementary material for: The Involvement of Melatonin in the Dimorphism of Glucose and Lipid Metabolism of Tilapia
Source: Biomolecules. 2025 Dec 21;16(1):15. doi: 10.3390/biom16010015 (PMC12838915; doi:10.3390/biom16010015)
Supplement: Supplementary file 1 [file Figure S5.pdf › Figure S5.pdf]

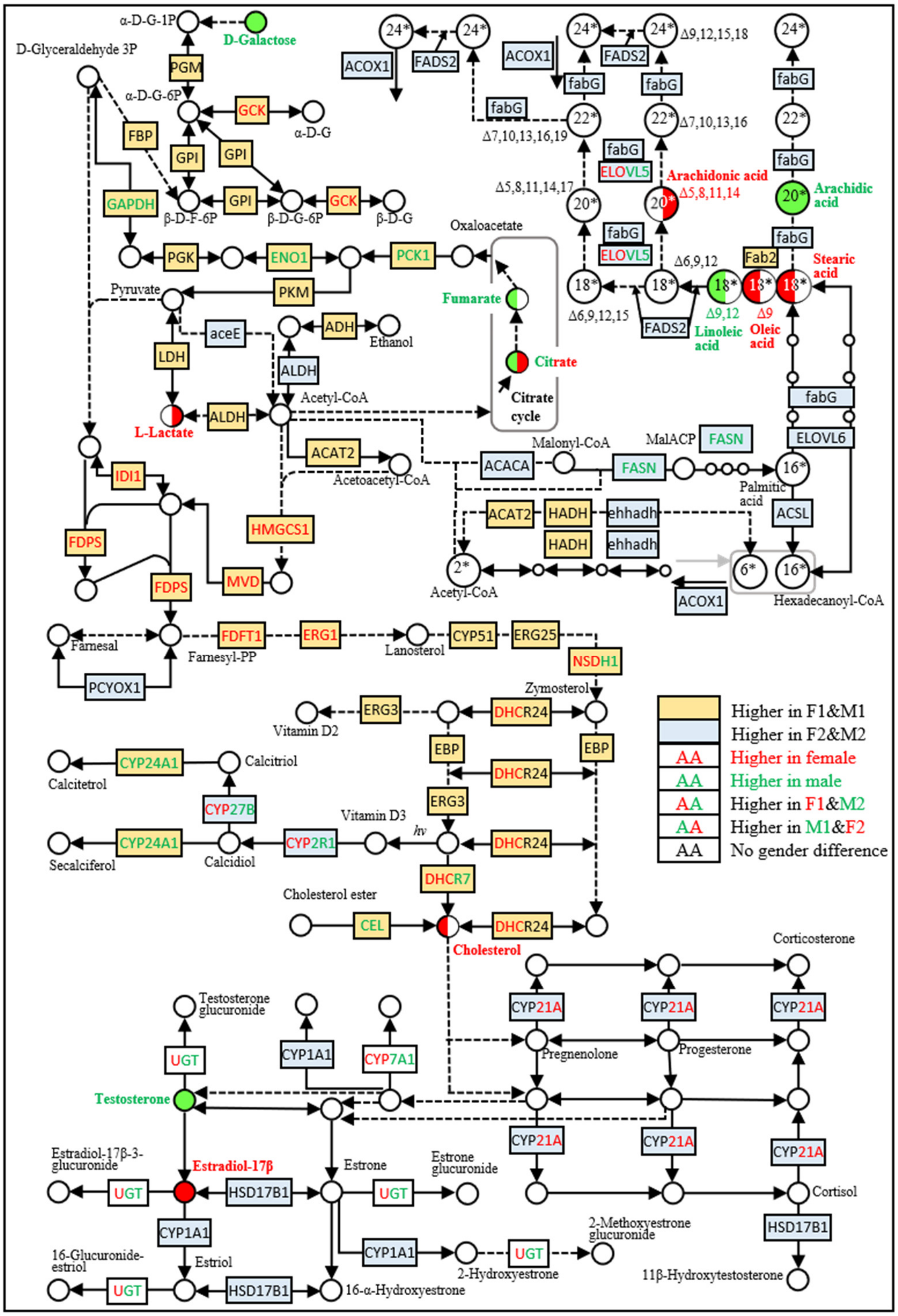

Figure S5. Integrated analysis of DGE and metabolomic profiles in glucose and lipid metabolic pathways.
